# Immunization with detoxified TNFα elicits neutralizing antibodies and ameliorates inflammatory shock and autoimmune arthritis in mice

**DOI:** 10.3389/fimmu.2026.1801223

**Published:** 2026-04-10

**Authors:** Wen-Ling Hsu, He Ren, Wei-Chiao Huang, Ramkumar T. Annamalai, Yumiao Zhang, Jonathan F. Lovell

**Affiliations:** 1 Department of Biomedical Engineering, University at Buffalo, State University of New York, New York, NY, United States; 2 School of Synthetic Biology and Biomanufacturing, State Key Laboratory of Synthetic Biology, Tianjin University, Tianjin, China

**Keywords:** autoimmune disease, inflammation, liposomal vaccine, nanoparticles, rheumatoid arthritis, therapeutic vaccine, TNFα

## Abstract

**Introduction:**

Tumor necrosis factor-⍺ (TNF⍺) is central to the pathogenesis of autoimmune and inflammatory diseases, and monoclonal antibody-based TNF⍺ inhibitors are amongst the most used biologics worldwide. As an alternative to exogenously administered antibodies, active immunization has been explored as a TNF⍺ neutralization strategy.

**Methods:**

We generated a murine TNF⍺ (mTNF⍺) Y87S point mutant expressed in *E. coli* that preserved the native trimeric structure while abrogating receptor binding. Using histidine-tag interactions, the antigen was displayed on the surface of immunogenic cobalt porphyrin-phospholipid (CoPoP) liposomes, which further attenuated TNF⍺ toxicity and enabled safe immunization. Immunization and protective efficacy were evaluated in murine models, including lipopolysaccharide/galactosamine-induced lethal shock and collagen-induced arthritis (CIA). Parallel studies were conducted with a similarly engineered human TNF⍺ (hTNF⍺) Y87S mutant.

**Results:**

Liposome display of TNF⍺ elicited significantly higher levels of neutralizing antibodies following immunization. In mice, immunization improved survival in the lethal shock model and ameliorated clinical symptoms and joint inflammation in the CIA model. The Y87S mutation similarly detoxified hTNF⍺ constructs, which could also be effectively displayed on CoPoP liposomes. Immunization with hTNF⍺ induced high-titer anti-TNF⍺ IgG and serum neutralizing activity superior to other adjuvants.

**Discussion:**

These findings demonstrate that CoPoP-based TNF⍺ point mutant vaccines can safely induce high levels of functional TNF⍺-neutralizing antibodies. This strategy represents a potential low-cost alternative to current TNF-blocking biologics for the treatment of autoimmune diseases.

## Introduction

Tumor necrosis factor α (TNFα) is a pleiotropic proinflammatory cytokine that plays a central role in orchestrating innate and adaptive immune responses. Dysregulated TNFα signaling contributes to the pathogenesis of numerous immune-mediated inflammatory diseases, including rheumatoid arthritis (RA), inflammatory bowel disease (IBD), psoriasis, plaque psoriasis, sarcoidosis, ankylosing spondylitis, and Behçet’s disease (1–5). These findings ignited the development of multiple disease-modifying antirheumatic drugs (DMARDs), most notably the FDA-approved TNFα inhibitors infliximab, adalimumab, golimumab, etanercept, and certolizumab pegol. Infliximab is a chimeric monoclonal antibody coupling human IgG1 with murine Fv regions of anti-human TNFα antibody, adalimumab and golimumab are fully human monoclonal antibodies, etanercept is a fusion protein of the soluble TNF receptor and Fc portion immunoglobulin and certolizumab pegol is a pegylated humanized anti-TNF Fab fragment (6, 7). By preventing TNFα from binding its receptors, these agents suppress inflammatory cascades and substantially improve clinical manifestations of RA, IBD, psoriasis, arthritis, and ankylosing spondylitis, particularly when combined with methotrexate (8–13).

Despite their transformative efficacy, TNFα inhibitors present certain potential limitations. Continuous blockade increases susceptibility to opportunistic infections, including upper respiratory infections, tuberculosis, and sepsis (14–20), and the relatively short serum half-life of these biologics (9–20 days) necessitate frequent intravenous or subcutaneous administration (6). Their high annual costs (up to US$18,000-26,000 per patient) also create substantial economic burden (21). Moreover, therapeutic responses are heterogeneous. Approximately two-thirds of patients fail to respond adequately to their first TNFα inhibitor within six months (22, 23). These challenges have sustained interest in innovative therapeutic strategies, including nanoparticle-based delivery systems, RNA interference approaches, and cytokine-targeted vaccines. Among these, the concept of an “anti-cytokine vaccine”, an immunogen designed to elicit durable polyclonal neutralizing antibodies against a pathogenic cytokine, was pioneered for TNFα and remains the most advanced example of active anti-cytokine immunotherapy (24, 25).

Nanoparticle-based interventions have shown particular promise. Acid-sensitive PEGylated solid-lipid nanoparticles carrying TNFα-siRNA significantly ameliorated symptoms in mouse collagen-induced arthritis (CIA) models (26). Synovium-targeted liposomes loaded with core peptide (a short nine amino acid immunosuppressant peptide derived from T-cell antigen receptor) reduced inflammation in rat adjuvant-induced arthritis (AIA) models (27). Efforts to develop TNFα vaccines have also been extensive. The TNFα kinoid (TNF-K), consisting of formaldehyde-inactivated human TNFα complexed with keyhole limpet hemocyanin (KLH), elicited high-titer neutralizing antibodies in hTNFα-transgenic (TTg) mice and protected against acute TNFα-induced shock and chronic arthritis (24, 25, 28). Although TNF-K safely induced anti-TNFα antibodies and produced modest clinical benefit, it did not meet its primary efficacy endpoint in a Phase IIb trial (29). Additional vaccine platforms, including papillomavirus virus-like particles (VLPs) displaying TNFα peptides (30), recombinant TNFα immunogens engineered with modified T-helper epitopes (31–33), and an epitope-scaffold TNF vaccine using diphtheria toxin transmembrane domain as a scaffold to present transmembrane TNF (34, 35) have demonstrated strong antibody induction and disease attenuation in murine models without provoking pathological T-cell activation.

Translating these encouraging preclinical results to humans remains challenging. Auto-vaccination against self-cytokine must overcome natural immunological tolerance while preserving the protective functions of endogenous TNFα required for pathogen control. Moreover, active cytokine vaccination poses distinct considerations relative to passive biologics. Immunogenicity depends on B-cell competence and may be attenuated by concomitant immunosuppression. Antibody titers and neutralizing capacity vary across individuals, and vaccine-induced immunity exhibits slower onset but potentially greater durability. These factors underscore the need for optimized antigen design, precision adjuvants, and delivery systems that can fine-tune antibody magnitude, specificity, and longevity.

Recent advancements in vaccine platforms could renew interest in anti-TNFα vaccination. Our group has developed cobalt porphyrin-phospholipid (CoPoP) liposomal vaccines for pathogens such as malaria (36–39), Lyme disease (40), and SARS-CoV-2 (COVID-19) (41, 42). CoPoP liposomes efficiently bind His-tagged antigens, are serum-stable and consistently induce strong, durable and balanced antibody responses. A CoPoP-based SARS-CoV-2 vaccine (EuCorVac-19) has successfully completed Phase 2 and 3 clinical trials (43–46). For the present study, we employed the CPQ adjuvant system, CoPoP combined with PHAD-3D6A (a synthetic monophosphoryl lipid A and TLR-4 agonist) and the saponin QS-21. PHAD-3D6A activates antigen-presenting cells in a non-pyrogenic manner (47, 48), and QS-21, synergistically enhances cytokine secretion and antibody production when co-formulated in the liposomal bilayer (49). Prior head-to-head comparisons showed that CP adjuvant outperformed commercial adjuvants such as Alum, Titer Max, AdjuPhos, QuilA, Sigma Adjuvant System, AddaVax (MF59-like), AS01, and Freund’s adjuvant (CFA/IFA) in eliciting high-titer antibody responses while maintaining low reactogenicity (36), and incorporation of QS-21 (CPQ) further elevated antigen-specific IgG titers and multifunctional CD4^+^ and CD8^+^ T-cells responses (39).

The aim of this study was to develop a therapeutic vaccine using CoPoP liposomes to overcome immune tolerance to TNFα and provide sustained protection against chronic inflammation. We developed a CoPoP-based TNFα vaccine to deliver a Y87S-mutated recombinant TNFα protein. The Y87S mutation preserves overall protein structure while attenuating cytotoxicity by disrupting TNFR1 binding. This antigen rapidly bound CoPoP liposomes and, upon vaccination, induced robust anti-mTNFα IgG titers and TNFα-TNFR1 neutralization. Immunization resulted in improved survival in the LPS/D-galactosamine-challenged mice and reduced disease severity in the CIA model. The analogous human Y87S mutant also resulted in attenuated cytotoxicity, and CoPoP immunization elicited neutralizing antibodies against hTNFα.

## Results

### Design and functional characterization of recombinant mTNFα

**Figure 1:**
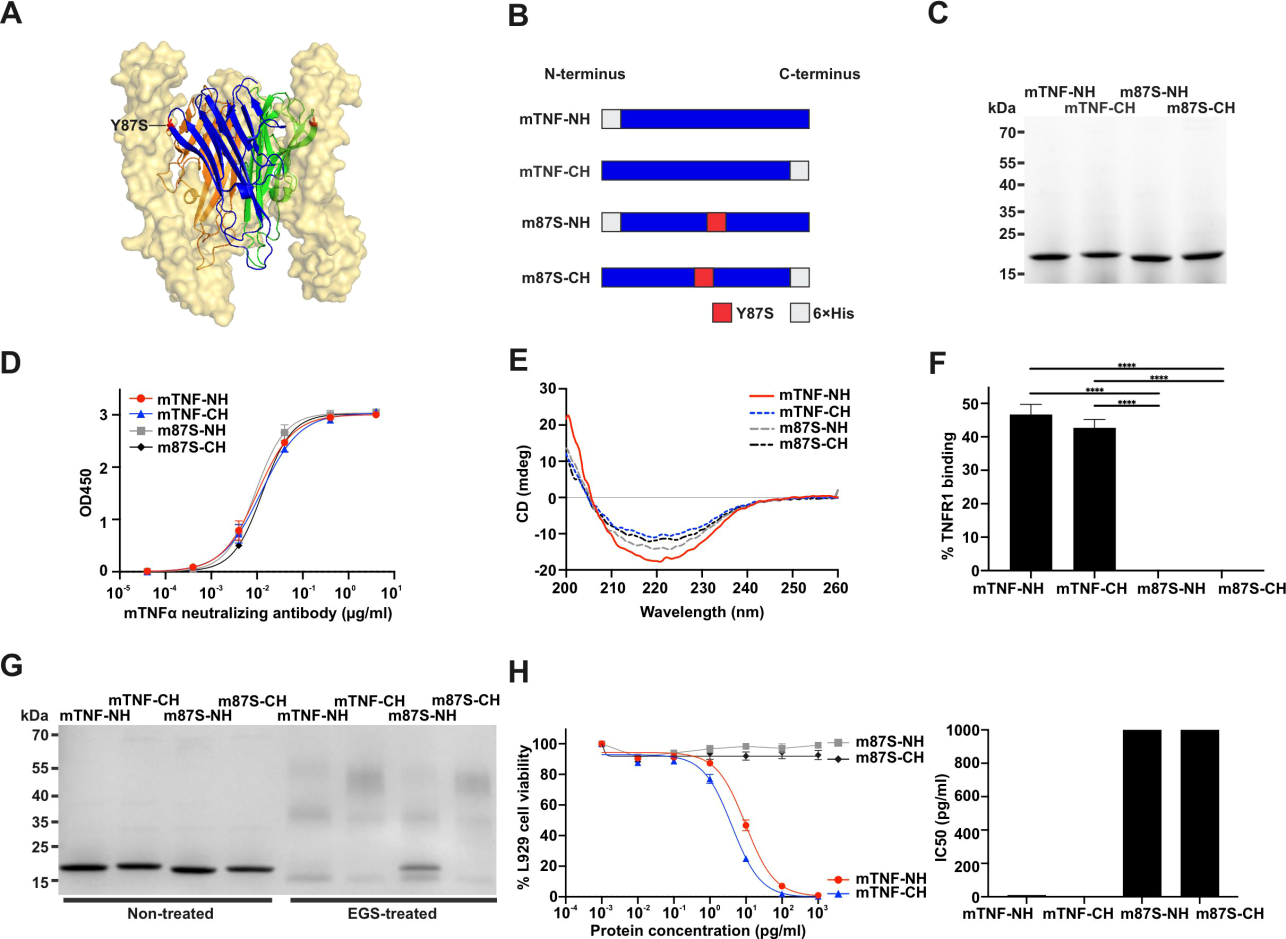
Characterization of recombinant mTNFα proteins and their functional properties. **(A)** Crystal structure of the mTNFα Y87S mutant trimer (ribbon representation) in complex with hTNFR1 (yellow spheres), generated using PyMOL (PDB code: 7KP7). The Y87S mutation site is highlighted in red. **(B)** Schematic representation of recombinant mTNFα constructs and site-directed mutations (red). mTNF-NH and mTNF-CH denote wildtype mTNFα with N- or C-terminal hexahistidine tags, respectively; m87S-NH and m87S-CH denote corresponding Y87S mutants. **(C)** SDS-PAGE analysis of purified wildtype and mutant mTNFα proteins. **(D)** ELISA confirmation of recombinant mTNFα identity. Immobilized proteins were detected by a monoclonal anti-mTNFα antibody in a dose-dependent manner. Data represent mean ± SD from triplicate measurements. **(E)** Circular dichroism spectra of mTNFα proteins collected at room temperature (0.3 mg/mL in water), showing far-UV signals from 200–260 nm. **(F)** Competitive binding assay showing inhibition of biotinylated mTNFα interaction with immobilized hTNFR1 by recombinant mTNFα proteins. Data represent mean ± SD from triplicate measurements. Statistical analysis was performed by one-way ANOVA followed by Tukey’s test. *p < 0.05, **p < 0.01, ***p < 0.005, and ****p < 0.001. **(G)** EGS cross-linking assay confirming trimer formation. Recombinant proteins (2.5 µg, 0.125 mg/ml) were incubated with 0.75 mM EGS (0.34 mg/ml) for 1 h at room temperature and analyzed by reducing SDS-PAGE. **(H)** L929 cytotoxicity assay and corresponding IC50 values for mTNFα proteins. IC50 values were obtained using a four-parameter logistic sigmoid model in GraphPad Prism. Data represent mean ± SD for n=3 measurements.

To enhance the soluble expression of recombinant murine TNFα (mTNFα), we introduced the Y87S point mutation, which has previously been reported to improve recombinant yield in human TNFα while substantially reducing cytotoxicity and receptor binding, yet preserving trimer formation and protein solubility (50). Notably, residue Y87 is conserved between the human and murine TNFα, supporting the rationale for applying this mutation to the murine protein. Structural modeling of the mTNFα mutant in complex with the human TNF receptor I (hTNFRI) trimers revealed that Y87 resides within the receptor interaction surface but does not participate in monomer-monomer interfaces (**Figure 1A**).

Wildtype mTNFα (mTNF-NH and mTNF-CH) and Y87S mutant proteins (m87S-NH and m87S-CH) were engineered with N- or C- terminal hexahistidine tags and expressed in *E. coli* (**Figure 1B**). All four constructs were produced at high yield (mTNF-NH: 1.35 mg; mTNF-CH: 1.02 mg; m87S-NH: 1.22 mg; m87S-CH: 1.4 mg) with good purity (**Figure 1C**) and were comparably recognized by a monoclonal anti-mTNFα antibody (**Figures 1C, D**). Circular dichroism spectroscopy confirmed preservation of secondary structure across all variants (**Figure 1E**).

Receptor binding was assessed using a competitive hTNFR1 binding assay. Whereas wildtype proteins retained more than 40% binding capacity, the Y87S mutants displayed no detectable receptor binding (**Figure 1F**). To determine whether this loss reflected impaired oligomerization, we performed EGS-mediated cross-linking. All constructs formed both trimers and dimers, with C-terminal His-tagged proteins yielding a higher proportion of trimeric species (**Figure 1G**). Functional activity was evaluated using L929 cytotoxicity assays, which revealed potent activity for wildtype mTNFα (IC50 = 9.754 and 4.088 pg/ml for mTNF-NH and mTNF-CH, respectively), whereas Y87S mutants exhibited IC50 values exceeding 1,000 pg/ml, indicating near-complete loss of cytotoxic function (**Figure 1H**).

To further examine structural features, we performed analysis using PyMOL based on the available TNFα-TNFR1 complex structure (7KP7) (51). In wildtype TNFα, residue Y87 forms a polar contact with A62 on TNFR1 at approximately 3.0 Å (**Supplementary Figure 1A**). This interaction is disrupted in the simulated Y87S mutant (**Supplementary Figure 1B**), which potentially explains the reduced receptor binding and cytotoxicity activity. In contrast, substitution with Y87S is more than 5 Å away from residues of adjacent TNFα monomer within the trimer (**Supplementary Figure 1C**), indicating that it does not participate in inter-monomer interactions. Accordingly, substitution with serine is not predicted to disrupt the trimeric interface (**Supplementary Figure 1D**). These structural predictions support our experimental findings that the Y87S mutation attenuates receptor engagement without affecting trimer assembly.

Together, these results demonstrate that the Y87S substitution preserves protein yield, folding, and trimerization while abolishing receptor binding and cytotoxicity, consistent with prior observations for human TNFα (50, 52), and support its use as a safe vaccine antigen.

### Characterization of liposome-bound mTNFα antigens

**Figure 2:**
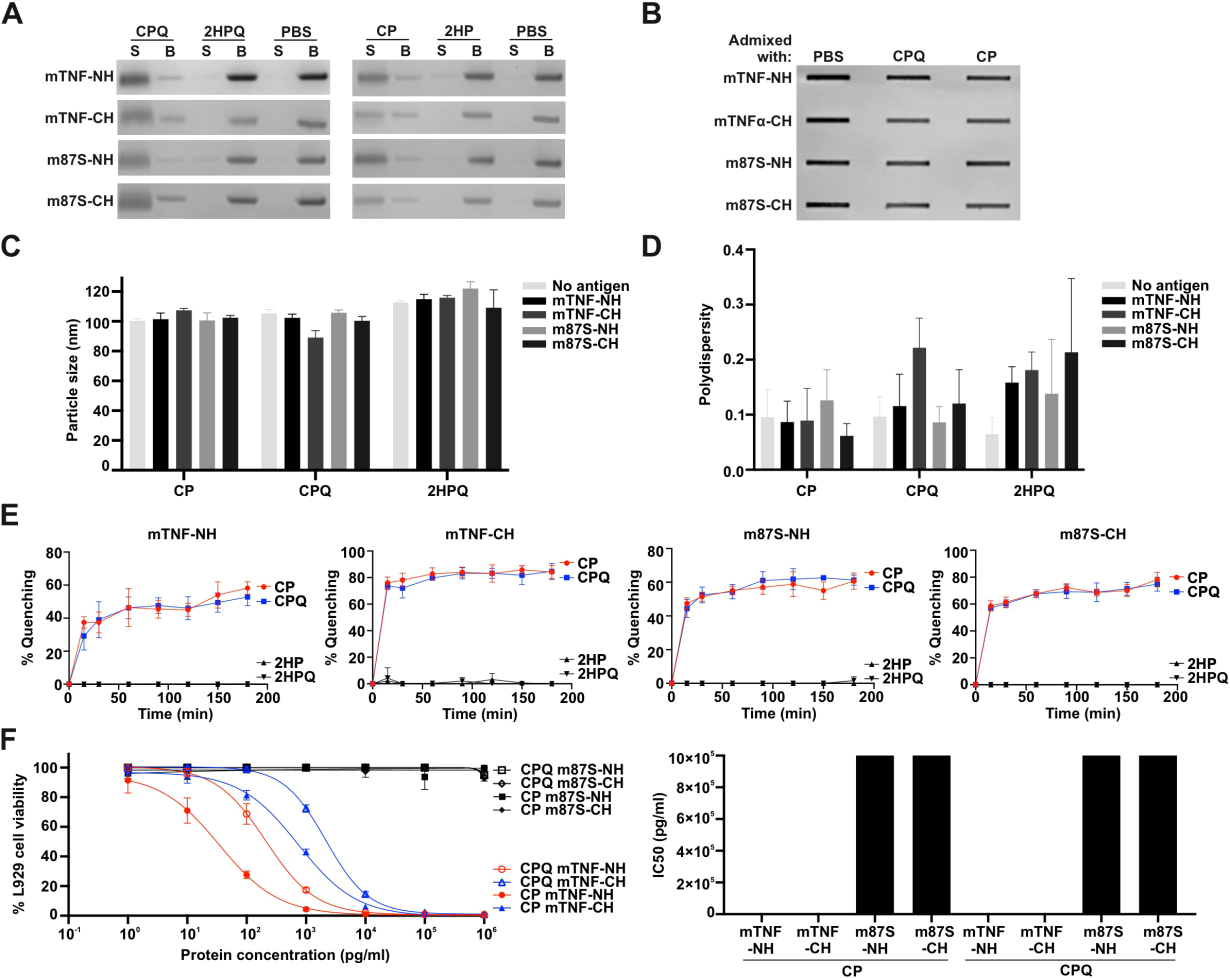
Characterization of CP and CPQ liposomes displaying recombinant mTNFα antigens. **(A)** Ni-NTA bead competition assay. Recombinant mTNFα proteins were incubated with liposomes for 3 h, after which Ni-NTA beads were added to capture unbound His-tagged proteins. Proteins retained on the beads (“B”) represent unbound soluble antigen, whereas liposome-associated proteins remained in the supernatant (“S”). **(B)** Slot blot detection of mTNFα protein bound to CoPoP liposomes using a monoclonal anti-mTNFα antibody. HRP signals were visualized by chemiluminescence. **(C)** Hydrodynamic size and **(D)** polydispersity index of liposomes following antigen binding, measured by dynamic light scattering (DLS). Data represent mean ± SD from triplicate measurements. **(E)** Kinetics of DY490-labeled mTNFα binding to liposomes, measured by fluorescence quenching. Data represent mean ± SD from triplicate measurements. **(F)** L929 cytotoxicity assay and corresponding IC50 values for liposome-displayed mTNFα proteins. IC50 values were calculated using a four-parameter logistic sigmoid model in GraphPad Prism. Data represent mean ± SD from triplicates measurements.

We next assessed the interaction of His-tagged mTNFα proteins with CP and CPQ liposomes. Ni-NTA bead competition assays demonstrated efficient binding of all constructs to both liposome formulations (**Figure 2A**). Slot blot analysis confirmed that antigenic integrity was preserved following liposome loading, as evidenced by strong recognition by an anti-mTNFα monoclonal antibody (**Figure 2B**).

Dynamic light scattering measurements showed that particle size remained within ~90–110 nm with low polydispersity after antigen loading (**Figures 2C, D**). Fluorescence quenching assays revealed rapid particle formation, with ~80% binding achieved for C-terminal His-tagged proteins and ~60% for N-terminal His-tagged proteins within 15 minutes (**Figure 2E**).

L929 cytotoxicity assays demonstrated substantial attenuation of biological activity upon liposome association. CP-bound wildtype mTNF-NH and mTNF-CH exhibited IC50 values of 34.55 and 736.9 pg/ml, respectively, whereas CPQ-bound proteins showed further attenuation (IC50 = 209.3 and 2,051 pg/ml). Liposome-bound Y87S mutants consistently exhibited minimal cytotoxicity (**Figure 2F**). These findings indicate that liposomal formulation reduces residual TNFα activity, an advantageous feature for vaccine safety.

### Liposome-bound mTNFα Y87S mutant elicits anti-mTNFα antibody responses in ICR mice

**Figure 3:**
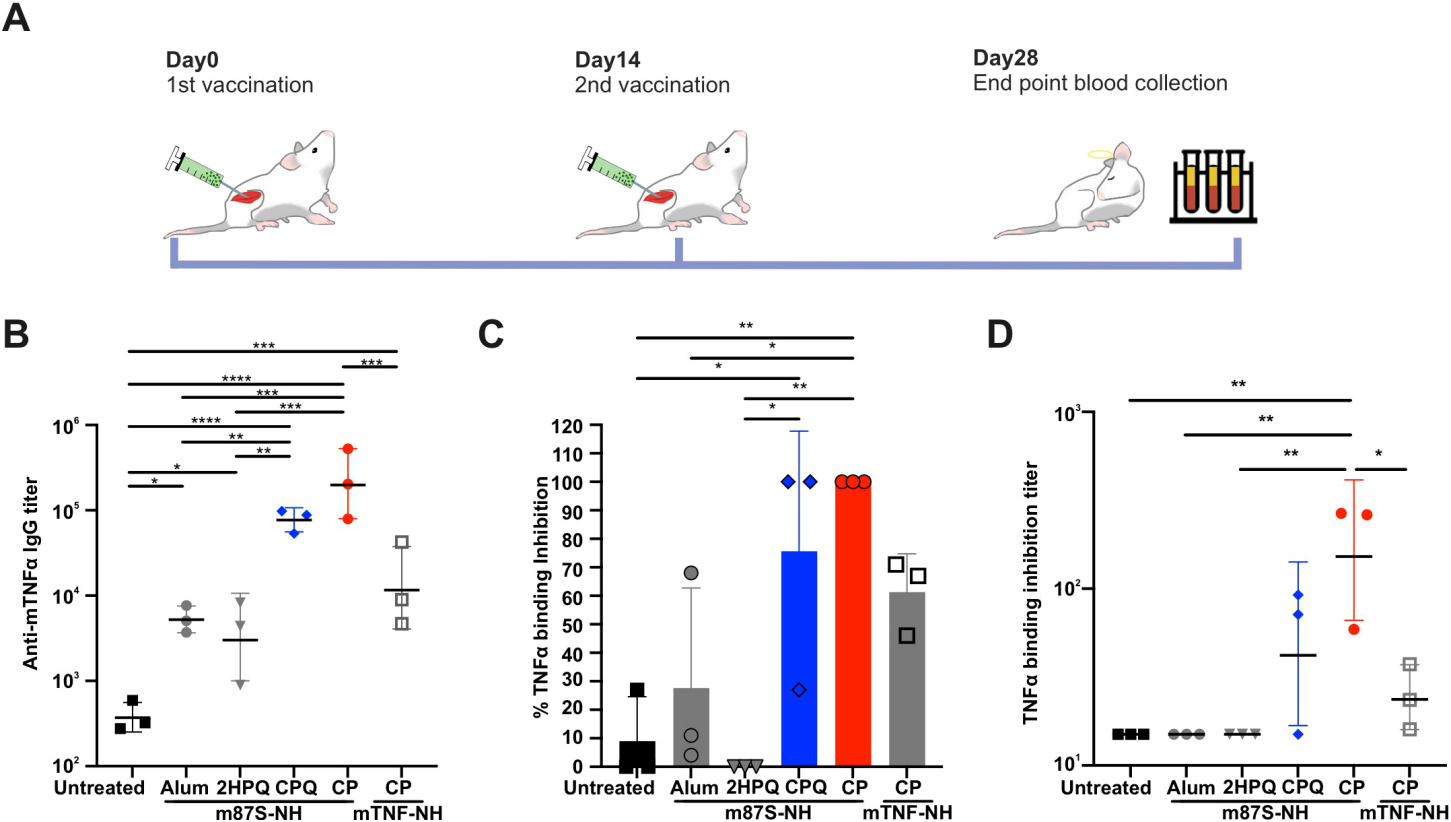
CP-m87S-NH vaccination induces antigen-specific antibodies that block mTNFα binding to TNF receptor. **(A)** Immunization scheme. ICR mice were immunized intramuscularly with mTNFα vaccines (2 µg antigen) on days 0 and 14, and serum was collected on day 28. **(B)** Anti-mTNFα IgG titer measured by ELISA. Groups included untreated (n=3), Alum/m87S-NH (n=3), 2HPQ/m87S-NH (n=3), CPQ/m87S-NH (n=3), CP/m87S-NH (n=3), and CP/mTNF-NH (n=3). **(C)** mTNFα-hTNFR1 binding inhibition (sera diluted 1:15 in PBS) and **(D)** corresponding inhibition titers. hTNFR1-coated plates were incubated with immunized serum pre-mixed with biotinylated mTNFα at 37 °C for 1 h. After transfer to the hTNFR1-coated plate and incubated for 30 minutes at 37 °C, unbound proteins were washed off with PBST. Captured biotinylated mTNFα was detected with streptavidin-HRP and TMB substrate. Lines and bar graphs show mean ± SD for n=3 mice per group. Statistical significance was determined by one-way ANOVA followed by Tukey’s test. *p < 0.05, **p < 0.01, ***p < 0.005, and ****p < 0.001.

To evaluate immunogenicity, outbred ICR mice were immunized intramuscularly on days 0 and 14 with CP-, CPQ-, 2HPQ-, or Alum-formulated mTNFα antigens (2 µg of m87S-NH or mTNF-NH per dose) (**Figure 3A**). Serum collected on day 28 revealed that CP-m87S-NH and CPQ-m87S-NH induced markedly higher anti-mTNFα IgG titers (2.7x10^5^ and 8x10^4^, respectively) compared with all other formulations (<1.9x10^4^) (**Figure 3B**).

Notably, sera from CP-m87S-NH-immunized mice most effectively inhibited mTNFα binding to hTNFR1, yielding the highest inhibition titer (2.2x10^2^) (**Figures 3C, D**). These findings demonstrate that CP-encapsulated m87S-NH is strongly immunogenic and induces functional antibodies capable of blocking TNFα-TNFR1 interactions *in vitro*.

### Neutralizing antibodies induced by CP-m87S-NH vaccination protect against LPS/D-galactosamine-induced lethal shock

**Figure 4:**
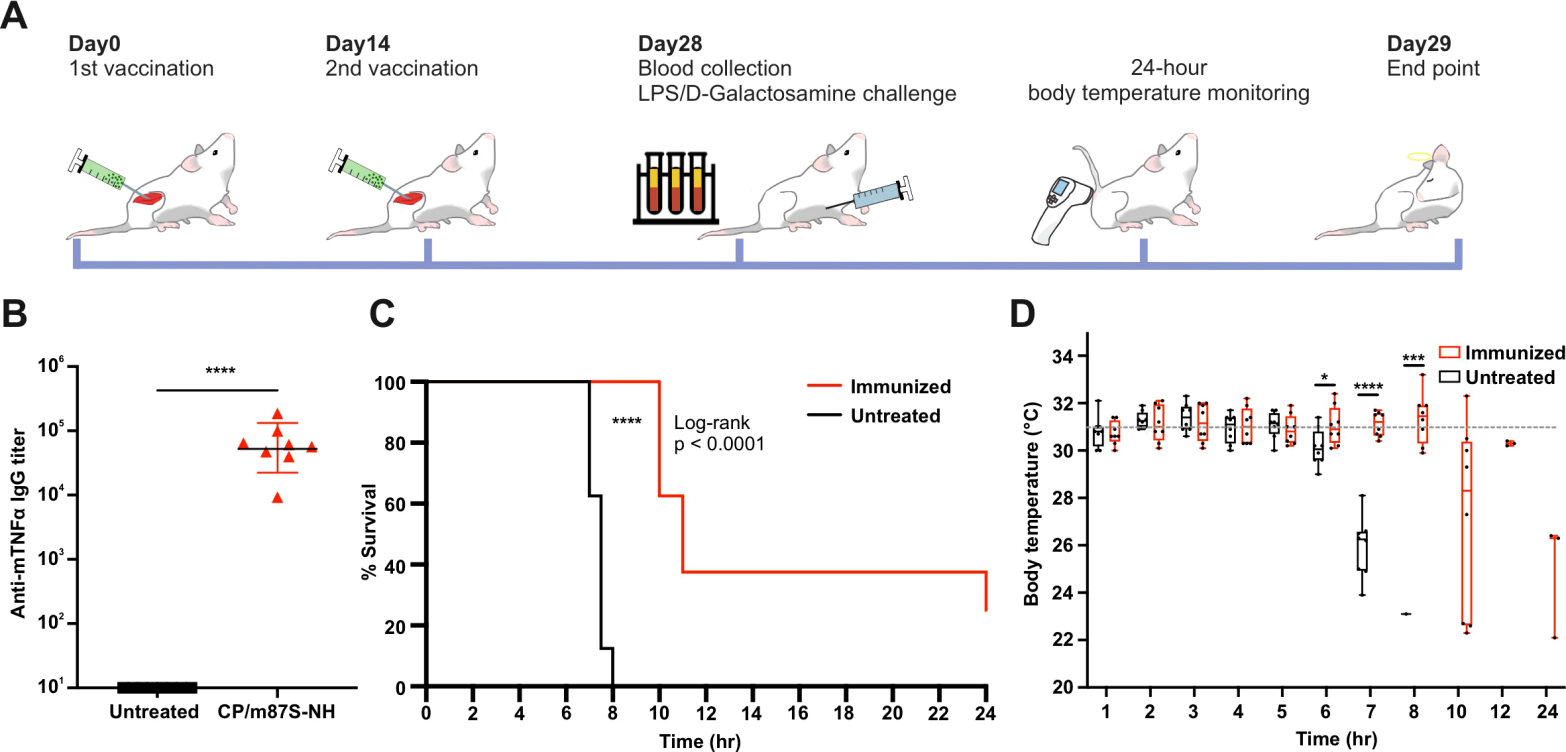
CP-m87S-NH vaccination protects mice from LPS/D-galactosamine-induced lethal shock. **(A)** Immunization and LPS/D-galactosamine challenge scheme. BALB/c mice were immunized intramuscularly with mTNFα vaccines (2 µg antigen) on days 0 and 14. Serum was collected on day 28, after which mice were injected intraperitoneally with LPS (1.5 ug) plus D-galactosamine (20 mg). Surface body temperatures were measured from the anogenital area using an infrared thermometer over 24 h. **(B)** Anti-mTNFα IgG titer measured by ELISA. Groups included untreated (n=8) and CP/m87S-NH (n=8). **(C)** Survival over 24 h post-challenge. **(D)** Body temperature profiles. Data are shown as mean ± SD (n=8 per group in the beginning). The body temperature baseline before the challenge is 30.98 °C (the dash line). Statistical significance for **(B, D)** was assessed by one-way ANOVA with Tukey’s test; *p < 0.05, **p < 0.01, ***p < 0.001, and ****p < 0.0001. Survival curves **(C)** were analyzed using Log-rank test; *p < 0.05, **p < 0.01, ***p < 0.001, and ****p < 0.0001.

We next investigated whether vaccine-elicited antibodies could neutralize TNFα activity *in vivo* using a LPS/D-galactosamine-induced lethal shock model (**Figure 4A**). BALB/c mice immunized with CP-m87S-NH developed high anti-mTNFα IgG titers (~10^5^) (**Figure 4B**). Following LPS/D-galactosamine challenge, CP-m87S-NH-immunuzed mice exhibited significantly improved survival compared with non-immunized controls, all of which succumbed within 8 hours (**Figure 4C**). Immunized mice also showed improved thermoregulation beginning 6 hours post-challenge (**Figure 4D**).

Although CP-m87S-NH did not confer complete protection, which is consistent with the multifactorial cytokine storm underlying this model, which is mediated by systemic secretion of pro-inflammatory cytokines, including TNFα, IL1, and IL6 and associated acute liver injury (53, 54). The observed partial protection indicates that vaccine-induced polyclonal antibodies can attenuate hyperinflammatory responses without fully suppressing physiological immune functions.

### CP-m87S-NH vaccination ameliorates disease severity in a murine CIA model

**Figure 5:**
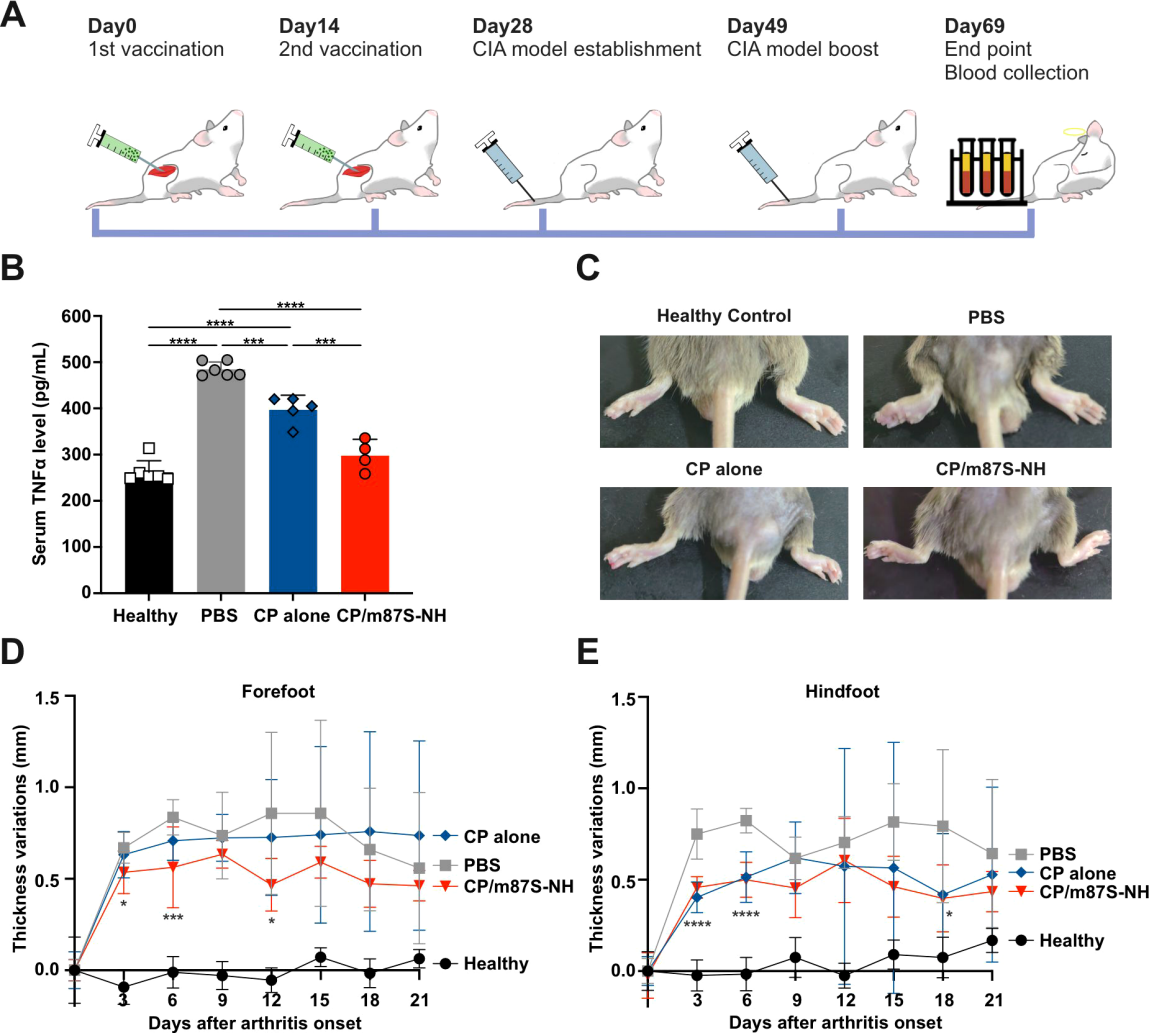
CP-m87S-NH vaccination ameliorates clinical symptoms in the murine CIA model. **(A)** Immunization and CIA induction scheme. Groups of 6 mice were immunized intramuscularly with mTNFα vaccines (2 µg antigen) on days 0 and 14. On day 28, the mice received an intradermal injection of CII emulsion and were boosted on day 49. Blood and limbs were collected for analyses on day 69. **(B)** Plasma TNFα levels. Groups included healthy controls (n=6), PBS (n=6), CP alone (n=5), and CP/m87S-NH (n=4). Data are shown as mean ± SD. **(C)** Representative hindfoot images. **(D)** Forefoot and **(E)** hindfoot thickness measurement over time. Data are shown as mean ± SD. Statistical significance for **(B)** was determined by one-way ANOVA followed by Tukey’s multiple-comparison test. Statistical significance between CP-m87S-NH and PBS controls in **(D,E)** was analyzed using two-way repeated-measures ANOVA with Geisser-Greenhouse correction followed by Dunnett’s multiple-comparison test, and P values were adjusted for multiple comparisons. *p < 0.05, **p < 0.01, ***p < 0.001, and ****p < 0.0001.

We next evaluated vaccine efficacy in the collagen-induced arthritis (CIA) model (**Figure 5A**). Mice were immunized intramuscularly with mTNFα vaccines (2 µg antigen) on days 0 and 14, followed by intradermal injection of bovine collagen II on day 28 and a booster on day 49. Blood and limb samples were collected on day 69, and foot swelling was monitored throughout disease progression.

CP-m87S-NH vaccination significantly reduced circulating TNFα concentrations (**Figure 5B**) and mitigated foot swelling, particularly during the first week following arthritis onset (**Figures 5C–E**). These results indicate that vaccination with CP-m87S-NH attenuates inflammatory pathology in experimental autoimmune arthritis.

### CP-h87S-NH vaccination induces neutralizing antibodies against human TNFα

**Figure 6:**
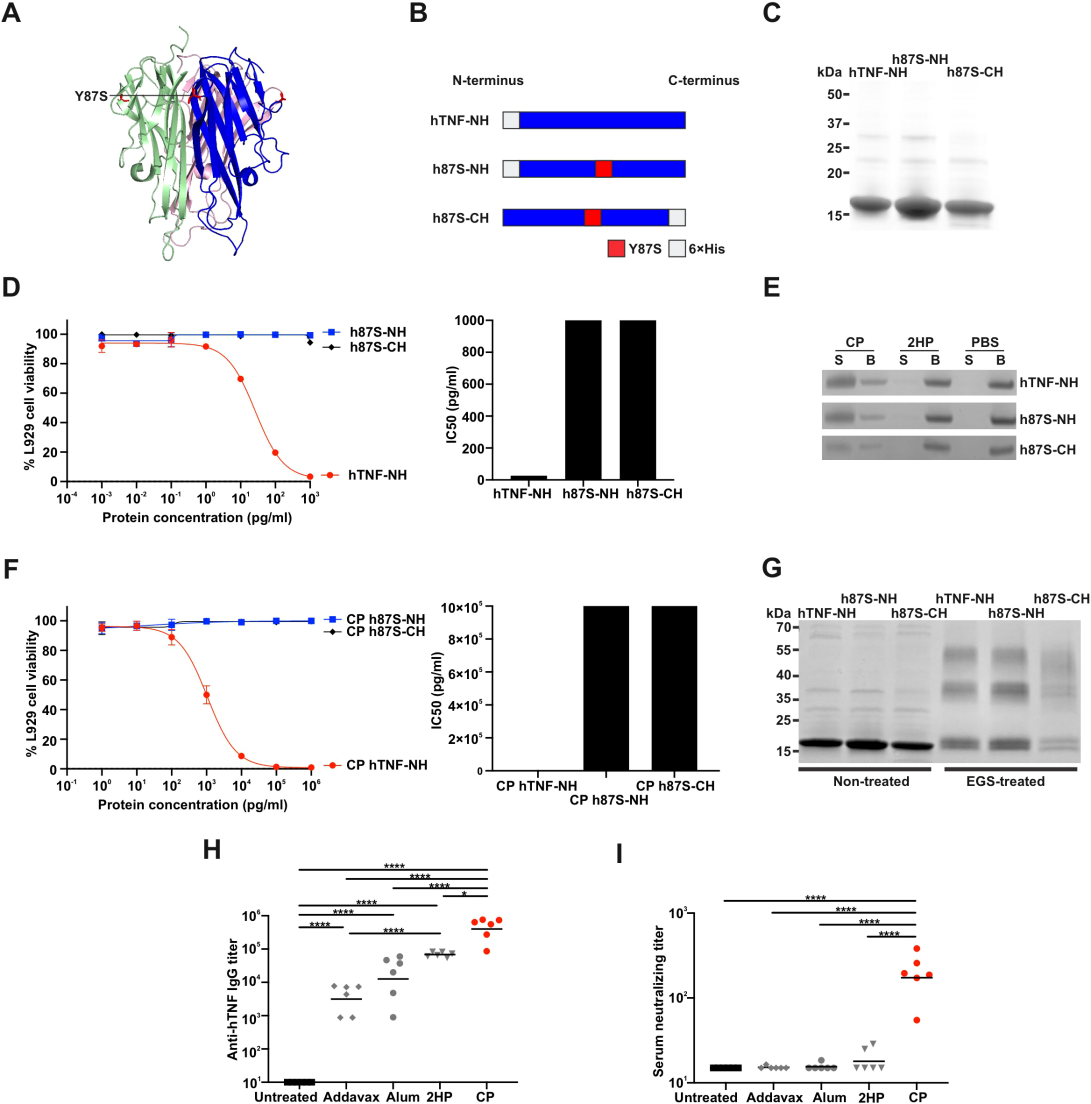
CP-h87S-NH vaccine elicits neutralizing antibodies against hTNFα. **(A)** Crystal structure of the hTNFα Y87S mutant trimer rendered in Pymol (PDB code: 1TNF), with the Y87S mutation highlighted in red. **(B)** Schematic of recombinant hTNFα constructs and site-directed mutations (red). hTNF-NH denotes wildtype hTNFα with a N-terminal His tag; h87S-NH and h87S-CH denote Y87S mutants with N- or C-terminal His tags. **(C)** SDS-PAGE analysis of purified wildtype and Y87S mutant proteins. **(D)** L929 cytotoxicity assay and IC50 values for hTNFα proteins, calculated using a four-parameter logistic sigmoid model (GraphPad Prism). Data represent mean ± SD for n=3 measurements. **(E)** Ni-NTA bead competition assay assessing liposome binding to recombinant hTNFα proteins. **(F)** L929 cytotoxicity assay and IC50 values for the liposome-encapsulated hTNFα proteins, calculated as in **(D)**. Data represent mean ± SD (n=3). **(G)** EGS cross-linking assay confirming trimer formation. **(H)** Anti-hTNFα IgG titers in collected on day 28 from ICR mice immunized intramuscularly with hTNFα vaccines (2 µg antigen) on day 0 and 14. Groups included untreated controls (n=6), AddaVax/h87S-NH (n=6), Alum/h87S-NH (n=6), 2HP/h87S-NH (n=6), and CP/h87S-NH (n=6). **(I)** Serum neutralizing titer measured by L929 assay. hTNFα protein was pre-incubated with diluted serum for 1 h before addition to L929 cells for a 20 h, followed by alamarBlue viability readout. Data represent mean ± SD (n=6 mice per group). Statistical significance was assessed by one-way ANOVA with Tukey’s test. *p < 0.05, **p < 0.01, ***p < 0.001, and ****p < 0.0001.

To extend these findings toward translational relevance, we produced recombinant wildtype human TNFα and Y87S mutant proteins (**Figures 6A, B**). All constructs were expressed at high yield (hTNF-NH: 1.66 mg; h87S-NH: 2.33 mg; h87S-CH: 1.15 mg) and good purity (**Figure 6C**). L929 cytotoxicity assays confirmed potent activity of wildtype hTNF-NH (IC50 = 26.4 pg/ml) and markedly reduced activity of Y87S mutants (IC50 > 1,000 pg/ml) (**Figure 6D**).

Ni-NTA competition assay revealed efficient binding of all constructs to CP liposomes (**Figure 6E**). Liposome association substantially attenuated activity, with CP-bound hTNF-NH showing IC50 = 1058 pg/ml and Y87S mutants exceeding 10^6^ pg/ml (**Figure 6F**). All proteins retained the ability to form dimers and trimers, as confirmed by EGS cross-linking (**Figure 6G**).

Immunization of ICR mice with CP-h87S-NH induced robust anti-hTNFα IgG titers (~5x10^5^), exceeding those elicited by all comparator formulations (<10^5^) (**Figure 6H**). Sera from CP-h87S-NH-immunized mice effectively neutralized hTNFα-induced cytotoxicity in L929 cells (**Figure 6I**). Together, these results establish CP-h87S-NH as a promising candidate for therapeutic vaccination against TNFα-mediated inflammatory diseases.

## Discussion

TNFα blockade has transformed the treatment landscape for autoimmune diseases such as rheumatoid arthritis, psoriatic arthritis, and inflammatory bowel disease. Despite their clinical success, monoclonal antibody biologics are associated with substantial limitations, including high manufacturing costs, laborious production pipelines, parenteral administration, immunogenicity against the therapeutic antibody, and the requirement for lifelong treatment (55, 56). A therapeutic vaccine strategy capable of eliciting long-lived neutralizing antibodies could mitigate these drawbacks by enabling sustained disease control through patient-generated humoral immunity (57–60).

In this study, we developed a particulate TNFα vaccine using a rationally engineered, nonfunctional Y87S TNFα mutant formulated within a CoPoP-containing liposomal platform. Structural modeling and biochemical analyses demonstrated that the Y87S substitution preserves the native trimeric architecture, which is essential for antigenicity, while abolishing TNFα cytotoxicity. This combination of structural preservation and functional inactivation is essential for a cytokine-targeted vaccine, as it presents native B-cell epitopes while avoiding harmful receptor mediated-signaling.

In the CIA model, CP-m87S-NH vaccination significantly attenuated disease severity, reduced foot swelling, and lowered circulating TNFα levels relative to PBS and adjuvant-only controls. These findings indicate that antibodies generated by the vaccine neutralize bioactive TNFα *in vivo*. Importantly, CP-m87S-NH conferred superior protection compared with CP alone, confirming that disease attenuation was antigen-specific rather than attributable to nonspecific adjuvant effects. Despite these improvements, the vaccine did not fully abrogate disease manifestations in the CIA model nor completely prevent lethality in the LPS/D-galactosamine challenge relative to healthy controls.

Only partial protection was observed in the LPS challenge model. This may reflect the stringent experimental conditions used and/or an insufficient neutralizing antibody response. Previous studies have shown that TNF knockout mice were completely protected from LPS/D-galactosamine-induced shock with LPS doses up to 100 µg (61), while administration of 100 µg etanercept protected mice challenged with 5 µg of LPS and 10 mg D-galactosamine (62). In our model, mice were challenged with 1.5 µg LPS and 20 mg D-galactosamine following a previously reported protocol (63). Although these studies demonstrate the effectiveness of genetic or pharmacological TNF inhibition, direct comparison with our results is limited due to differences in experimental design and challenge conditions. Future studies could evaluate vaccine efficacy using optimized immunization regimens, such as higher dosing, additional adjuvants, additional booster immunizations, and should include controls such as TNFα antibodies to benchmark vaccine efficacy.

The limited protection observed in the LPS challenge model reflects its multifactorial pathophysiology. In addition to soluble TNFα-mediated shock, this model induces hepatocyte apoptosis and lung injury driven by cell-associated TNFα, interleukin-17A (IL17A) (64, 65), CC chemokine receptor 9 (CCR9) (66), and CC chemokine ligand 25 (CCL25) (67). Because the vaccine targets soluble TNFα and does not interfere with upstream pathways or cell-bound cytokines, it is expected to mitigate but not eliminate pathology (68). Consistent with this interpretation, previous therapeutic vaccine studies have demonstrated that targeting IL17A and IL-23p19 can improve experimental arthritis outcomes (69, 70). These observations support the rationale for future optimization strategies, including antigen dose refinement and the incorporation of additional inflammatory targets such as IL-17A, CCR9, CCL25, and IL-23p19 to enhance therapeutic breadth and efficacy. Moreover, advanced micro-computed tomography and histopathological analyses may be incorporated into future studies to further evaluate vaccine safety and elucidate protective mechanisms in the CIA model.

Although binding of vaccine-induced antibodies to transmembrane TNFα (tmTNFα) was not directly evaluated in this study, the antigen design suggests that the elicited polyclonal antibodies may recognize both soluble TNFα (sTNFα) and tmTNFα, as the vaccine antigen contains the extracellular domain of TNFα (residues 77-233) shared by both forms. Clinically used TNFα inhibitors, including infliximab, adalimumab, and etanercept, are known to bind tmTNFα on cell surfaces (71, 72), although generally with lower affinities than for sTNFα (73). Selective modulation of tmTNFα has been reported to influence inflammatory responses. For example, anti-tmTNFα polyclonal antibodies increased tmTNFα expression while reducing sTNFα production and partially protected mice from LPS-induced septic shock (74), whereas mice expressing uncleavable tmTNFα exhibit resistance to endotoxin-induced shock (75). At the same time, strong inhibition of tmTNFα has been associated with increased susceptibility to opportunistic infections during anti-TNF therapies (76). Taken together, vaccine-induced polyclonal antibodies are expected to predominantly neutralize sTNFα while potentially exhibiting partial reactivity toward tmTNFα. Compared with monoclonal antibody therapies, this polyclonal response may provide more moderate TNFα inhibition, which could reduce the risk of excessive immunosuppression. Future studies will be needed to directly evaluate antibody binding and functional effects on tmTNFα-expressing cells.

Some previous TNFα vaccine studies using the CIA model have employed prophylactic immunization prior to arthritis induction (30, 31, 33, 77, 78), primarily to evaluate the ability of vaccination to overcome immune tolerance and generate neutralizing anti-TNFα antibodies before disease onset. This represents an initial efficacy assessment, since TNF inhibitors are administered therapeutically after arthritis onset. For example, etanercept (30 mg/kg) administered after disease onset in the CIA model demonstrated stronger anti-inflammatory effects than TNF vaccination in that study (77). A separate study using the DTNF7 vaccine reported therapeutic efficacy when vaccination was initiated after CIA induction (34). A limitation of the present study is that we evaluated only the prophylactic efficacy of the vaccine. Future studies should therefore assess therapeutic vaccination initiated after symptom onset and directly compare vaccine efficacy with injectable TNF inhibitors.

To explore translational potential, we next evaluated human TNFα vaccine constructs. Comprehensive biochemical characterization, including SDS-PAGE, cytotoxicity assays, liposome competition assays, and trimer cross-linking verified proper expression, purification, folding, and compatibility with the CP delivery platform. Immunization with CP-h87S-NH induced robust anti-TNFα IgG titers and superior functional neutralization relative to conventional adjuvants, including Alum, AddaVax and 2HP. These results highlight the importance of the CP platform in promoting strong and functionally relevant humoral immunity against TNFα.

A major concern associated with anti-TNFα therapies is the increased susceptibility to bacterial, viral, fungal, and opportunistic infections, and screening for latent tuberculosis is routinely required prior to treatment initiation (76). Monoclonal antibodies such as infliximab and adalimumab can bind tmTNFα with high affinity and may trigger complement-dependent cytotoxicity and antibody-dependent cellular cytotoxicity, potentially leading to depletion of immune cells involved in host defense. In contrast, etanercept exhibits weaker binding to tmTNFα and reduced complement recruitment, which may contribute to a lower risk of immune cell depletion (71, 79). Previous TNFα vaccine studies provide some insights into the safety profile of this approach. For example, vaccination with a KLH-conjugated TNF vaccine using full-length soluble murine TNFα did not impair immune responses against intracellular pathogens such as *Listeria monocytogenes* and *Mycobacterium tuberculosis* compared with etanercept treatment (77). Another study using a virus-like particle TNF vaccine reported that immunization with a TNF peptide antigen did not affect immunity against intracellular bacteria, whereas immunization with full-length sTNFα increased susceptibility (30). Interestingly, the peptide formulation exhibited weaker binding to tmTNFα and provided less protection in the CIA arthritis model compared with the full-length antigen. These findings suggest that the long-term immunological consequences of TNFα vaccination may depend on antigen design and adjuvant selection. For clinical translation, further studies would be required to evaluate long-term safety, infection susceptibility, and potential reversibility of vaccine-induced anti-TNFα immunity.

Collectively, these findings establish CP-based TNFα vaccination as a promising therapeutic strategy that integrates precision antigen engineering with an immunostimulatory particulate delivery system optimized for durable antibody production. This approach has the potential to overcome key limitations of biologic TNFα inhibitors, offering a long-acting and cost-effective alternative for the treatment of chronic autoimmune diseases. The findings also underscore the importance of structural fidelity in cytokine vaccine design and provide a foundation for next-generation strategies that incorporate additional inflammatory targets. Future studies will focus on dose optimization, durability of immune protection, safety assessment in additional species, and the potential integration of this platform with complementary immunomodulatory targets.

## Conclusion

Our findings demonstrate that rationally engineered TNFα Y87S antigens, delivered using the CP particulate platform, safely elicit potent TNFα-neutralizing antibodies. The Y87S substitution preserves the native trimeric structure required for immunogenicity while abolishing receptor binding and cytotoxicity. The CP-m87S-NH vaccine mitigates disease severity in the murine CIA model, while the humanized CP-h87S-NH formulation induces stronger neutralizing responses than traditional adjuvants. Together, these results highlight a promising and cost-effective vaccine strategy that could serve as an alternative to monoclonal TNFα inhibitors for treating autoimmune diseases and pave the way for multivalent therapeutic vaccines targeting multiple cytokines for better efficacy.

## Materials and methods

### Recombinant TNFα protein expression and purification

The wildtype TNFα sequences used for our studies were the soluble chains of TNFα proteins from mouse (aa80-235) and human (aa77-233), retrieved from UniProtKB/Swiss-Prot database (accession numbers: P06804 and P01375). The TNFα mutant candidates were selected and modified from the residue with the highest yield and solubility listed in a previous report (50). The pET-3a plasmids containing our engineered constructs were manufactured by GenScript (Piscataway, NJ). The BL21 (DE3) *E. coli* strain was obtained from Intact Genomics (MO, USA). The protein expression construct included a 6-histidine tag at the C- or N-terminus of the protein. Full amino acid sequences are shown in **Supplementary Table 1**.

An *E. coli* DirectPlate™ BL21 (DE3) chemically competent cell clone (Intact Genomics, Cat# 1019-36) harboring the target plasmid was streaked on a LB plate (Teknova, Cat# L1093) with 100 μg/mL Ampicillin (Quality Biological, Cat# 351-344-731) and incubated at 37 °C for 16 hours. A single colony was selected and grown in 2 mL of trypticase soy broth (BD Diagnostics, Cat# 292770) supplemented with 100 μg/mL Ampicillin for 3 hours. The resulting culture was inoculated into 50 mL of terrific broth (Gibco, Cat# A1374301) with 100 μg/mL Ampicillin and incubated at 37 °C until OD_600_ reached 1.0-1.2. Isopropyl-*β*-D-thiogalactopyranoside (IPTG) (Invitrogen, Cat# 15529019) was added to a final concentration of 1 mM for 3 hours of induction at 37 °C. The cell pellet was collected after centrifugation at 4,000g for 30 minutes.

The pellet was lysed by incubation in B-PER Complete Bacterial Protein Extraction Reagent (Thermo Scientific, Cat# PI89822) according to the manufacturer’s instructions. The lysate was centrifuged at 16,000g for 20 minutes to remove cell debris, and the supernatant was collected for further protein purification steps. The lysate was mixed with 1X volume of Binding Buffer (50 mM Na_2_HPO_4,_ 300 mM NaCl, 10 mM Imidazole, pH 8.0). A total of 0.3 mL of Ni-NTA resin (Thermo Scientific, Cat# 88221) was packed into a 5 mL column, rinsed with 1 mL of deionized H_2_O, and equilibrated with 1 mL of Binding Buffer. The lysate was then added to the column and passed through it ten times. After washing with 25 mL of Wash Buffer (50 mM Na_2_HPO_4,_ 300 mM NaCl, 20 mM Imidazole, pH 8.0), the target protein was eluted by adding 0.6 mL of Elution Buffer (50 mM Na_2_HPO_4,_ 250 mM NaCl, 300 mM Imidazole, pH 8.0) three times. The eluted fractions were collected and dialyzed against 1 L of PBS three times. Lastly, the purified proteins were filtered through a 0.22 μm membrane and examined using SDS-PAGE analysis. Protein concentrations were determined by using a BCA assay.

### Characterization of the recombinant TNFα proteins

Characterization of the recombinant TNFα proteins were assessed by ELISA using 96-well plates (Thermo Scientific, Cat# 442404) coated with 1 μg/mL of recombinant TNFα proteins in coating buffer (3.03g Na_2_CO_3_; 6 g NaHCO_3_ in 1 L deionized water, pH 9.6) for 1 hour at 37 °C. After washing and blocking with 2% BSA in PBS containing 0.1% Tween-20 (PBST), serial dilutions of anti-mouse TNFα monoclonal antibody (eBioscience, Cat# 14-7321-81) in 2% BSA were added and incubated for 1 hour at 37 °C. After washing with PBST, HRP-linked anti-mouse IgG antibody (Cell Signaling, Cat# 7076S) was added, followed by 3,3’,5,5’-tetramethylbenzidine (TMB) substrate (Surmodics, Cat# TMBW-1000-01). The reaction was stopped with 1M HCl, and absorbance was measured at 450 nm.

### Circular dichroism analysis

Structural characterizations of the recombinant TNFα proteins were measured on a circular dichroism spectrophotometer (JASCO Inc, Cat# J-1700) at room temperature. Far-UV spectra from 260 to 200 nm were measured on a TNFα sample of 0.3 mg/mL in DI H_2_O. The parameter: quartz cuvette path = 1 mm, bandwidth = 1 nm, data pitch = 0.1 nm, scanning speed = 20 nm/min.

### TNFRI receptor binding competition assay

Measurement of the recombinant TNFα proteins binding to TNRF1 were assessed by ELISA using 96-well plates coated with 50 ng/well of human TNFR1 protein (ACROBiosystems, Cat# TN1-H5222) in coating buffer for 1 hour at 37 °C. After washing and blocking with 2% BSA in PBST, 50 ng/well of TNFα proteins were added and incubated for 1 hour at 37 °C. After washing with PBST, 50 ng/well of biotinylated mouse TNFα protein (ACROBiosystems, Cat# TNA-M82E9) was added and incubated for 30 min at 37 °C. After washing with PBST, 100 μL of HRP-conjugated streptavidin (Thermo Scientific, Cat# N100) was added (1:2500 dilution) and incubated for 1 hour at 37 °C. After washing with PBS-T, 3,3’,5,5’-tetramethylbenzidine (TMB) substrate (Surmodics, Cat# TMBW-1000-01) was added. The reaction was stopped with 1M HCl, and absorbance was measured at 450 nm.

### TNFα trimerization assay

Recombinant TNFα proteins were diluted in DPBS to 0.125 mg/mL, and 20 μL aliquots were treated with the cross-linker ethylene glycolbis(succinimidylsuccinate) (EGS, 16.1Å) (Thermo Scientific, Cat# 21565), and the final concentration of the cross-linker was 0.34 mg/ml. The reaction was carried out at room temperature for 1 h, and the aliquot from each group was analyzed by reducing SDS-PAGE and visualized by SimplyBlue SafeStain (Invitrogen, Cat# LC6065).

### TNFα activity bioassay

The TNFα activity was assessed by using the L929 cytotoxicity assay (24). L929 cells (ATCC, Cat# CCL-1) were cultured in triplicates in 96-well cell culture plates (Greiner Bio-One, Cat# 655180) at 4 × 10^3^ cells/well for 18 h. Serial dilutions of TNFα were incubated with the cells for 18 h in the presence of actinomycin D (1 μg/mL) (Gibco, Cat# 11805017) and the number of surviving cells were determined by the alarmaBlue assay (Bio-Rad, Cat# BUF012B). The percentage of L929 viability was calculated by the following formula:


$$\%\;Viability\;=\;\frac{Fluorescence\;at\;590\;nm\;of\;the\;test\;agent\;}{Fluorescence\;at\;590\;nm\;of\;the\;untreated\;control}\;\times \;100$$

### Liposome preparation

Liposomes were prepared using ethanol injection and nitrogen-pressurized lipid extrusion as reported previously (36). The chemicals used for liposome synthesis are 1,2-dioleoyl-sn-glycero-3-phosphocholine (DOPC) (Corden, Cat# LP-R4-070), cholesterol (PhytoChol) (Wilshire Technologies, Cat# 57-88-5), Monophosphoryl Hexa-acyl Lipid A, 3-Deacyl (PHAD-3D6A) (Avanti Polar Lipids, Cat# 699855), QS-21 (Desert King), CoPoP and porphyrin-phospholipid (PoP) were produced as described previously (80, 81). The prepared liposomes were dialyzed against PBS twice at 4 °C to remove ethanol and passed through a 0.2 μm sterile filter. The final liposome concentration was adjusted to 320 μg/mL and stored at 4 °C. Liposome sizes and polydispersity index were determined by dynamic light scattering (DLS) using a NanoBrook 90 plus PALS instrument after 500-fold dilution in PBS. The CoPoP/PHAD (CP) liposomes had a mass ratio of [DOPC: CHOL: CoPoP: PHAD-3D6A] [20:5:1:0.4]. PoP/PHAD-3D6A (2HP) liposomes, which contain hydrogen instead of cobalt in the PoP, served as control liposomes, with a mass ratio of [DOPC: CHOL: PoP : PHAD-3D6A] [20:5:1:0.4]. For liposomes containing QS-21, QS-21 (1 mg/mL) was added to the liposomes after formation at an equal mass ratio as PHAD-3D6A. The CoPoP/PHAD-3D6A/QS-21 (CPQ) had a mass ratio of [DOPC: CHOL: CoPoP: PHAD-3D6A:QS-21] [20:5:1:0.4:0.4], PoP/PHAD-3D6A/QS-21 served as control liposomes with the same mass ratio of [DOPC: CHOL: PoP : PHAD-3D6A:QS-21] [20:5:1:0.4:0.4].

### Ni-NTA bead competition assay

To assess recombinant protein binding stability in particle form, Ni-NTA magnetic beads (ThermoFisher, Cat# 88831) were added to the liposome-incubated antigens (1:4 mass ratio of total protein: CoPoP) or free proteins in PBS. The samples were incubated with the beads for 30 minutes at room temperature. Following incubation, the supernatant and beads were separated using a magnetic separator (ThermoFisher, Cat# 12321D). The beads were then resuspended in PBS, and denaturing reducing loading dye was added to all samples (supernatant and beads), followed by heating at 95 °C for 10 minutes. A total of 46 μL samples (1.5 μg protein) were loaded into each well of a Tris-Glycine gel (Bio-Rad, Cat# 4568084). PageRuler Prestained Protein Ladder (4 μL) (Thermo Scientific, Cat# 26616) was also loaded. Gels were run at a constant 200 V for 30 minutes and stained with SimplyBlue SafeStain (Invitrogen, Cat# LC6065) following the manufacturer’s instructions. Gel images were acquired using a Bio-Rad GelDoc Go Imaging System.

### Slot blot assay

A 0.2 μm nitrocellulose membrane (Thermo Scientific, Cat# 88013) was affixed in a 48-well microfiltration apparatus (Bio-Rad, Cat# 170–6545). Each well was first loaded with 50 μL of PBS, followed by 50 μL of sample (50 ng), which flowed through the membrane by gravity. After blocking the membrane with 5% bovine serum albumin (VWR, Cat# 97061-422) for 1 hour at room temperature, the membrane was incubated with anti-mouse TNFα monoclonal antibody (eBioscience, Cat# MP6-XT22) at a 1:1000 dilution in 2% BSA for 1 hour at 37 °C. The membrane was washed three times with PBS, followed by incubation with HRP-linked anti-mouse IgG antibody (Cell Signaling, Cat# 7076S) at a 1:1000 dilution in 2% BSA for 1 hour at 37 °C. The membrane was washed again with PBS and treated with VisiGlo HRP substrate mixture (VWR, Cat# 97063–148). Images were captured using a Bio-Rad ChemiDoc Imager.

### Fluorescence quenching assay

The recombinant protein (200 μg) was dialyzed into 100 mM sodium bicarbonate buffer (pH 9.0) using a Slide-A-Lyzer Dialysis Cassette (Thermo Scientific, Cat# 66383) twice at 4 °C. The protein was labeled with 10 μL of 1 mg/mL DY-490-NHS-Ester (Dyomics, Cat# 490-01) for 1 hour at room temperature with continuous mixing. The labeled protein was then dialyzed with PBS three times to remove excess dye. The fluorescence of the labeled protein (491 nm excitation, 527 nm emission) was measured using a TECAN Safire microplate reader to assess binding efficiency to liposomes. Upon binding to liposomes, fluorescence was quenched due to energy transfer from DY-490-TNFα to the porphyrin in CP/CPQ or 2HP/2HPQ (36). Binding kinetics were monitored by the changes in fluorescence after incubating the same volume of protein (80 μg/mL) with liposomes in PBS (320 μg/mL) or PBS alone. Samples were diluted 10-fold prior to measurement in the plate reader. The binding percentage was calculated using the following equation:


$$\%\text{Binding}=1-(\frac{{\text{Fluorescence}}_{\text{test liposomes}}}{{\text{Fluorescence}}_{\text{free labeled protein}}})\times 100\%$$

### Murine immunization

Murine studies were performed according to protocols approved by the University at Buffalo or Tianjin University IACUC. Three strains of mice were used for different studies. Six- to eight-week-old female ICR (CD-1) mice (Charles River, Strain code 022) and BALB/c mice (Charles River, Strain code 028) were used for neutralizing antibody titer measurement and LPS/D-galactosamine-induced shock. DBA/1J mice (TIANJIN GUOSHENG ZHONGYUAN TECHNOLOGY CO., LTD., Cat# 11005A) were used for the CIA study. For immunization, the mice received intramuscular injections on days 0 and 14 containing 2 μg TNFα proteins combined with the liposomal adjuvants CP, CPQ or 2HPQ liposomes. The commercial adjuvant Alum (InvivoGen, Cat# vac-alu-50) was used as a comparison. CPQ vaccines were prepared by incubating TNFα protein (80 μg/mL) with liposomes (320 μg/mL) for 3 hours at room temperature. Alum vaccines were prepared by mixing antigen with Alum diluted to 1 mg/mL in PBS. On day 28 post-immunization, blood was collected from mice under 5% isoflurane anesthesia, followed by humane euthanasia via cervical dislocation. Sera were separated by centrifuging the whole blood at 2000g for 20 minutes at 4 °C.

### Antigen-specific antibody titer measurement

Anti-TNFα IgG titers were assessed by ELISA using 96-well plates coated with 1 μg/mL of TNFα and blocked with 2% BSA in PBST. Serial dilutions of mouse sera in PBS were added and incubated for 1 hour at 37 °C. After washing with PBST, HRP-linked anti-mouse IgG antibody (Cell Signaling, Cat# 7076S) was added, followed by 3,3’,5,5’-tetramethylbenzidine (TMB) substrate (Surmodics, Cat# TMBW-1000-01). The reaction was stopped with 1M HCl, and absorbance was measured at 450 nm. Titers were defined as the reciprocal serum dilution at which the absorbance exceeded the background by 0.5 absorbance units.

### Competition assay between immunized sera and TNFα receptor

To evaluate the presence of neutralizing antibodies in the immunized sera, we used a competition assay to detect the antibodies blocking the interaction between TNFα and its receptor. ELISA plates were coated with 50 ng/well of human TNFR1 protein (ACROBiosystems, Cat# TN1-H5222) for 1 hour at 37 °C. 50 μl of sera (1:15 dilution) were incubated with 50 μl of biotinylated mouse TNFα protein (10 ng) (ACROBiosystems, Cat# TNA-M82E9) for 1hour at room temperature. After washing and blocking with 2% BSA in PBST, 100 μl of the mixture was transferred to each well and incubated for 30 min at 37 °C. After washing with PBST, 100 μL of HRP-conjugated streptavidin (Thermo Scientific, Cat# N100) was added (1:2500 dilution) and incubated for 1 hour at 37 °C. After washing with PBST, TMB substrate (Surmodics, Cat# TMBW-1000-01) was added. The reaction was stopped with 1M HCl, and absorbance was measured at 450 nm. The inhibition percentage was calculated using the following equation:


$$\%\;Inhibition\;=(1\;-\;\frac{OD450\;of\;the\;tested\;sample}{OD450\;of\;the\;negative\;control})\times 100$$

The inhibition titer was expressed as the reciprocal of the serum dilution inhibiting 50% of the TNFα binding.

### LPS/D-galactosamine-induced lethal shock

8 BALB/c mice were preimmunized with the CP/mouse TNFα liposomes, and a group of 8 mice were untreated control group. Two weeks after the boost, the mice were injected intraperitoneally with 1.5 µg lipopolysaccharide (LPS, *E. coli* O111:B4) (Sigma-Aldrich, Cat# L4391) together with 20 mg of D-galactosamine hydrochloride (Sigma-Aldrich, Cat# G0500) in sterile PBS. The mice were monitored for 24 h. Body temperature and body weight were recorded. Humane endpoints were set at: 1) > 20% body weight loss, 2) significant hypothermia (surface temperature below 24.3 °C, 3) severely unkept coat appearance, 4) fully hunched posture, 5) lethargic activity, 6) severely dehydration, or 7) irregular slow/shallow or fast/labored respiration.

### Collagen-induced arthritis model

Male DBA/1J mice were used to establish the CIA model. Mice were immunized intramuscularly with mTNFα vaccines (2 µg antigen) on days 0 and 14. Bovine type II collagen (1 mL) (Chondrex, Cat# 20022) was mixed with an equal volume of complete Freund’s adjuvant (CFA) (Chondrex, Cat# 7001) on ice to prepare the CII emulsion. Emulsion stability was verified by placing a drop into a beaker of water and a stable emulsion remained as an intact clump without dispersing. On day 28, mice were immunized intradermally at the base of the tail with 100 μL of the CII emulsion. On day 49, a booster immunization consisting of the same amount of type II collagen emulsified in incomplete Freund’s adjuvant (IFA) (Chondrex, Cat# 7002) was administered intradermally. Blood and limbs were collected for analyses on day 69. The foot swelling was monitored since the arthritis started.

### Serum TNFα level measurement

On day 69, blood was collected from CIA mice mentioned above, and serum TNF-α concentrations were quantified using a commercial ELISA kit (LunChangShuo Biotech, Cat# ED-20852) according to the manufacturer’s instructions.

### Serum neutralizing assay

The ability of immunized mouse serum to neutralize TNFα activity was similarly determined after incubating sera with hTNFα in the TNFα activity bioassay. The immunized mouse sera were serial diluted and incubated with 2,000 pg/ml of the homemade recombinant hTNFα in the presence of actinomycin D (1 μg/mL) (Gibco, Cat# 11805017) for 1 h at 37 °C prior adding to L929 cells. The positive control wells were added with 100 ng of the anti-mouse TNF⍺ monoclonal antibody (eBioscience, Cat# 14-7321-81). The L939 cells were incubated with the mixture for 18 hours at 37 °C until cell viability determination. The neutralizing titer was expressed as the reciprocal of the serum dilution that neutralized 50% of hTNFα activity.

## Data Availability

The original contributions presented in the study are included in the article/**Supplementary Material**. Further inquiries can be directed to the corresponding authors.
