# Supplementary material for: Immunization with detoxified TNFα elicits neutralizing antibodies and ameliorates inflammatory shock and autoimmune arthritis in mice
Source: Front Immunol. 2026 Apr 10;17:1801223. doi: 10.3389/fimmu.2026.1801223 (PMC13106428; doi:10.3389/fimmu.2026.1801223)
Supplement: Supplementary file 1 [file Table1.docx]

Supplementary Material of

Immunization with detoxified TNFα elicits neutralizing antibodies and ameliorates inflammatory shock and autoimmune arthritis in mice

Wen-Ling Hsu^1†^, He Ren^2†^, Wei-Chiao Huang^1^, Ramkumar T. Annamalai^1^, Yumiao Zhang^2*^, Jonathan F. Lovell^1*^

^1^Department of Biomedical Engineering, University at Buffalo, State University of New York, New York, USA.

^2^School of Synthetic Biology and Biomanufacturing, State Key Laboratory of Synthetic Biology, Tianjin University, Tianjin, China.

^†^These authors have contributed equally to this work and share first authorship.

*** Correspondence:***Yumiao Zhang: ymzhang88@tju.edu.cn

*Jonathan F. Lovell: [jflovell@buffalo.edu](mailto:jflovell@buffalo.edu)

**This PDF file includes:**

Supplementary Figures 1

Supplementary Table 1


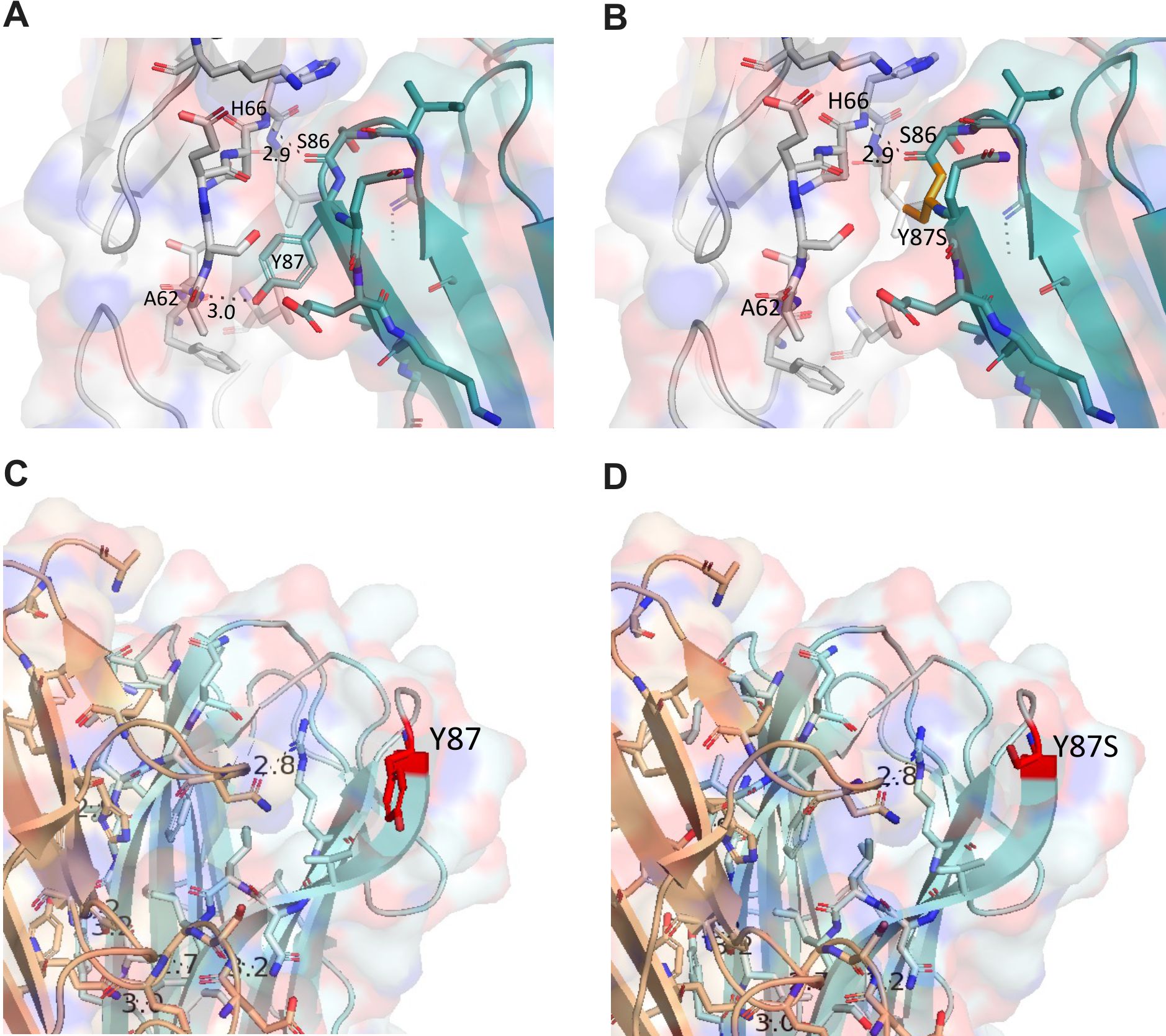


**Supplementary Figure 1: Predicted three-dimensional structure of TNFα trimers and TNFR1.** Side view of trimeric mTNFα bound to three copies of monomeric TNFR1 (PDB: 7KP7). **(A)** Interface between wildtype TNFα (green) and TNFR1 (gray). The Y87 residue on TNFα forms a polar contact (3.0 Å) with residue A62 on TNFR1. **(B)** This polar interaction is disrupted after the Y87S point mutation (orange). **(C)** Interface between two wildtype TNFα monomers (orange and blue). The Y87 residue (red) on one TNFα is located >5 Å from residues of the adjacent monomer, indicating no direct interaction. **(D)** This lack of interaction between TNFα monomers is preserved after the Y87S mutation (red).

**Supplementary Table 1: Sequences used in this study**

| Construct | Sequence |
| --- | --- |
| mTNF-NH | HHHHHH-LRSSSQNSSDKPVAHVVANHQVEEQLEWLSQRANALLANGMDLKDNQLVVPADGLYLVYSQVLFKGQGCPDYVLLTHTVSRFAISYQEKVNLLSAVKSPCPKDTPEGAELKPWYEPIYLGGVFQLEKGDQLSAEVNLPKYLDFAESGQVYFGVIAL |
| mTNF-CH | LRSSSQNSSDKPVAHVVANHQVEEQLEWLSQRANALLANGMDLKDNQLVVPADGLYLVYSQVLFKGQGCPDYVLLTHTVSRFAISYQEKVNLLSAVKSPCPKDTPEGAELKPWYEPIYLGGVFQLEKGDQLSAEVNLPKYLDFAESGQVYFGVIAL-HHHHHH |
| m87S-NH | HHHHHH-LRSSSQNSSDKPVAHVVANHQVEEQLEWLSQRANALLANGMDLKDNQLVVPADGLYLVYSQVLFKGQGCPDYVLLTHTVSRFAIS**S**QEKVNLLSAVKSPCPKDTPEGAELKPWYEPIYLGGVFQLEKGDQLSAEVNLPKYLDFA ESGQVYFGVIAL |
| m87S-CH | LRSSSQNSSDKPVAHVVANHQVEEQLEWLSQRANALLANGMDLKDNQLVVPADGLYLVYSQVLFKGQGCPDYVLLTHTVSRFAIS**S**QEKVNLLSAVKSPCPKDTPEGAELKPWYEPIYLGGVFQLEKGDQLSAEVNLPKYLDFAESGQVYFGVIAL-HHHHHH |
| hTNF-NH | HHHHHH-VRSSSRTPSDKPVAHVVANPQAEGQLQWLNRRANALLANGVELRDNQLVVPSEGLYLIYSQVLFKGQGCPSTHVLLTHTISRIAVSYQTKVNLLSAIKSPCQRETPEGAEAKPWYEPIYLGGVFQLEKGDRLSAEINRPDYLDFAESGQVYFGIIAL |
| h87S-NH | HHHHHH-VRSSSRTPSDKPVAHVVANPQAEGQLQWLNRRANALLANGVELRDNQLVVPSEGLYLIYSQVLFKGQGCPSTHVLLTHTISRIAVS**S**QTKVNLLSAIKSPCQRETPEGAEAKPWYEPIYLGGVFQLEKGDRLSAEINRPDYLDFAESGQVYFGIIAL |
| h87S-CH | VRSSSRTPSDKPVAHVVANPQAEGQLQWLNRRANALLANGVELRDNQLVVPSEGLYLIYSQVLFKGQGCPSTHVLLTHTISRIAVS**S**QTKVNLLSAIKSPCQRETPEGAEAKPWYEPIYLGGVFQLEKGDRLSAEINRPDYLDFAESGQVYFGIIALHHHHHH |
